# Variability and population structure of watermelon mosaic virus in zucchini crops in Poland

**DOI:** 10.1099/jgv.0.002273

**Published:** 2026-07-03

**Authors:** Daria Budzyńska, Julia Minicka, Aleksandra Zarzyńska-Nowak, Martyna Szkatulska, Agnieszka Taberska, Beata Hasiów-Jaroszewska

**Affiliations:** 1 Department of Virology and Bacteriology, Institute of Plant Protection-National Research Institute, Poznań, 60-318, Poland

**Keywords:** phylogenetics, potyviruses, survey, watermelon mosaic virus (WMV), zucchini

## Abstract

Watermelon mosaic virus (species *Potyvirus citrulli*, WMV) poses a significant threat to various cucurbit species worldwide. In Poland, the virus has been consistently recorded in *Cucurbita pepo* var. *giromontiina* crops since 2008. To analyse the genetic diversity and population structure of WMV in Poland, comprehensive surveys were conducted in two major agricultural regions, Greater Poland and Kuyavian–Pomeranian Voivodeships (west-central and mid-northern regions of Poland, respectively). To better understand the WMV occurrence, over 1,800 leaf samples were randomly collected from symptomatic and asymptomatic zucchini and weed plants. Moreover, aphids were also analysed for the presence of the virus. In this study, 154 complete coat protein gene sequences of WMV isolates collected between 2013 and 2024 were obtained using Sanger or high-throughput sequencing. The sequence identity of the Polish WMV isolates ranged from 92.8 to 100% and 95.4 to 100% for nucleotide and amino acid sequences, respectively. Unique mutations were identified in the isolates originating from the Kuyavian-Pomeranian region. Moreover, a novel KEET motif was identified in nine isolates, which has not been reported in previous studies. Most CP codons undergo negative selection; however, eight positively selected sites were detected. Phylogenetic analysis revealed that the Polish isolates were grouped in both classical and emergent main phylogenetic clades. The population analyses indicated limited or no gene flow between WMV subpopulations, suggesting that genetic drift may be considered an important evolutionary force shaping the viral diversity.

## Data availability

The sequences have been deposited in GenBank under accession nos. PV665942–PV666095, and Sequence Read Archive (SRA) data are available under BioProject accession no. PRJNA1237398.

## Introduction

Commercially cultivated members of the *Cucurbitaceae* family belong to the group of major economically important vegetables worldwide. In 2024, global cucurbits production included ~105 megatonnes (Mt) of watermelon (*Citrullus lanatus*), ~87 Mt of cucumbers and gherkins, ~34 Mt of pumpkins, squashes and gourds (*Cucurbita* spp.) (Food and Agriculture Organization of the United Nations, FAOSTAT Statistical Database, 2024). Cucumbers, together with pumpkins, squashes and gourds (including zucchini), are the predominant grown cucurbit species in Poland. Cucurbit crops are known to be susceptible to a variety of diseases, pests and environmental stresses that can impact their growth and productivity. At least 90 virus species representing the major plant virus genera were described to infect cucurbit crops [1]. It was estimated that plant viruses, together with viroids, cause annual losses of $30–50 billion in cultivated and stored crops and, consequently, represent a substantial barrier to the effective production and distribution of food [2].

Potyviruses (genus *Potyvirus*) represent the largest group of plant-infecting RNA viruses and belong to the family *Potyviridae*, which comprises 13 genera, distinguished based on host range, genomic features or phylogeny, with *Potyvirus* being the most species-rich among them. The first potyviruses, such as lettuce mosaic virus (species *Potyvirus lactucae,* LMV) and potato virus Y (species *Potyvirus yituberosi,* PVY), were identified in the 1920s/1930s [3, 4]. Their number has significantly increased with advances in diagnostic methods. From 16 species recognized in 1959 [5], the total rose to 57 by 1991 [6] and currently stands at 238 species, according to the ICTV report (21 April 2026). Potyviruses comprise a diverse group of species with a broad host range. Most are transmitted by aphids and, to a lesser extent, by seeds, and cause significant losses in yield and quality across a wide range of crops worldwide.

Previous studies have demonstrated that potyviruses share a common evolutionary origin with another genus within the *Potyviridae* family, *Rymovirus*. These groups constitute sister taxa that diverged from a common ancestor. Even though their genes and proteins are very similar, small genetic differences cause noticeable changes in how they coexist in the environment, especially in how they spread, for example, through aphids or mites [7]. Previous studies have concluded that recombination is an important factor driving the evolution of potyvirus populations. Although recombination does not frequently lead to the emergence of new species, several recombinant viruses have been reported, such as watermelon mosaic virus (species *Potyvirus citrulli*, WMV), which is considered a probable recombinant between soybean mosaic virus (species *Potyvirus glycitessellati*, SMV) and bean common mosaic potyvirus (species *Potyvirus phaseovulgaris*, BCMV) [7,8]. Owing to their agricultural importance, potyviruses have become major subjects of study and have provided important insights into virus biology and virus–environment interactions. The main potyviruses affecting cucurbits include WMV, zucchini yellow mosaic virus (species *Potyvirus cucurbitaflavitesselati*, ZYMV) and papaya ringspot virus (species *Potyvirus papayanuli*, PRSV) [1,9,11]. All of them belong to the group of viruses transmitted by aphids, the most common and highly efficient vectors of plant viruses [12].

The research concerning WMV isolates dates back to the 1950s/1960 [13,14]. First, based on the serological properties and host range, the virus isolates were divided into two different groups: WMV-1 and WMV-2. Later experiments led to the conclusion that WMV-1 is closely related to other potyvirus member, PRSV, and therefore, according to recent knowledge, it is classified as the W strain of this virus [15,16]. In Poland, WMV was detected almost half a century later, in 2008 [17]. Since then, several reports have confirmed WMV occurrence in both single and mixed infections with other viruses such as cucurbit aphid-borne yellows virus (species *Polerovirus CABYV*, CABYV), cucumber mosaic virus (species *Cucumovirus CMV*, CMV) or ZYMV [18,20], and it has been recently found that WMV seems to be the most widespread cucurbit virus in Poland [21]. WMV is a single-stranded, positive-sense RNA virus with a genome of ~10 kb in length and consists of flexuous, filamentous particles 750 nm long [17,22]. The ~9.6 kb ORF encodes a polyprotein, further processed by three viral proteases into ten putative functional proteins, with an 11th protein expressed through translational frameshift [23]. WMV has a wide experimental and natural host range, including watermelon, melon, cucumber, pumpkin, amaranth and sesame [15,19]. On infected plants, WMV can cause both mild and severe symptoms in the form of discolouration, deformations of leaves and fruits [17,24, 25]. WMV also infects many weeds that can serve as alternative hosts, but generally, weeds do not display symptoms of infection [10].

In recent years, the current epidemiological and phylogenetic status of WMV has been investigated worldwide [10,26]. WMV isolates were divided based on the coat protein gene (*cp*) sequences into three main phylogenetic groups: G1, G2 (both referred to as CL – classical strain, mainly grouping isolates obtained before 2000), and G3 (referred to as EM – emergent strain characterized by the newest isolates causing more severe symptoms in infected plants) [27,28]. The accessibility of high-throughput sequencing (HTS) techniques over the past decade has revolutionized plant virology, enabling large-scale studies on the occurrence of specific viruses, including WMV, and highlighting evolutionary dynamics of viruses infecting cucurbits [29].

In this study, the first comprehensive analysis of the diversity and population structure of WMV from zucchini crops in Poland was carried out. The results revealed that the Polish WMV population is well structured and highly diverse. The analysis allowed us to identify unique mutations in the isolates originating from the Kuyavian–Pomeranian region. Moreover, a novel KEET motif was identified in nine isolates, which has not been reported in previous studies. According to the phylogenetic analysis, the Polish isolates belong to both classical (CL) and emergent (EM) main WMV phylogenetic clades.

## Methods

### Sample collection

During the 2022–2024 growing seasons, samples of zucchini (*Cucurbita pepo* var. *giromontiina*), weeds and aphids were collected from cultivation fields located in the Greater Poland (west-central) and Kuyavian–Pomeranian (mid-northern) Voivodeships of Poland and were examined for WMV infection.

**Fig. 1.**
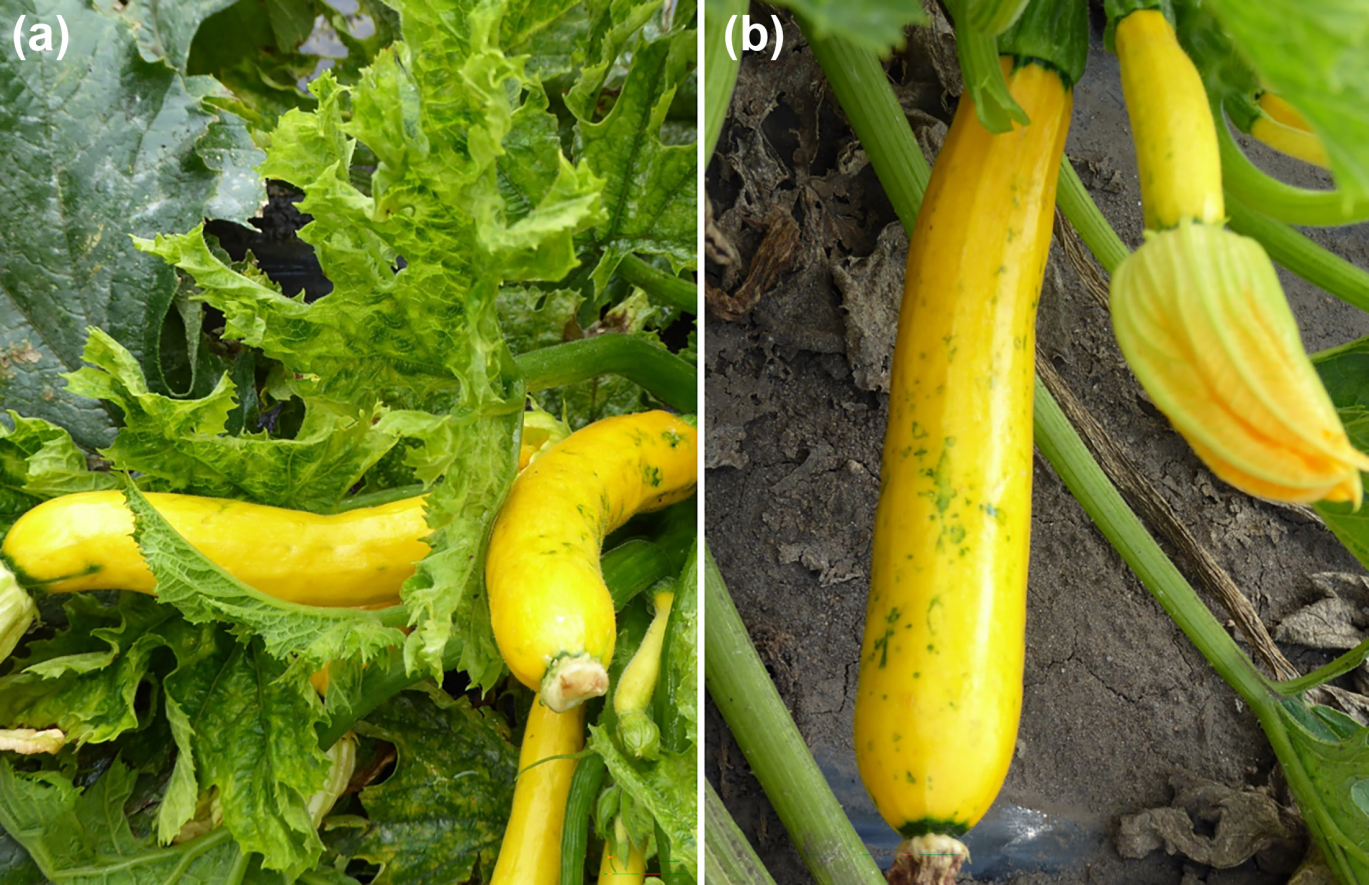
Symptoms on *C. pepo* var. *giromontiina* plants infected by the Polish WMV isolates. (a) Chlorotic mosaic and leaf deformation. (b) Deformation, mosaic and discolouration of fruits.

To get random and representative samples, plants were collected along a transect with 10-m intervals, sampling zucchini and other forbs (i.e. weeds) associated with each new point along the transect. Two to three young leaves were collected per plant. In total, samples were obtained from over 1,800 plants. Zucchini plants exhibiting virus-like symptoms, including chlorotic mosaic, deformation and discolouration of leaves and fruits (Fig. 1), as well as asymptomatic plants were collected. Weed samples represented various species from different families, such as *Amaranthaceae*, *Geraniaceae* and *Caryophyllaceae*, with most showing no obvious symptoms of viral infection. Furthermore, in the course of the study, 49 zucchini samples collected before 2022, belonging to the collection of the Department of Virology and Bacteriology and Laboratory of the Plant Diseases Clinic and Bank of Pathogens of IPP-NRI (Poznań, Poland), were analysed.

To monitor the appearance of the first aphid individuals, at least two yellow traps were installed in each sampling field. Also, the direct inspection of zucchini plants and other hosts was carried out at the beginning of the growing season, combined with the sampling of aphids. Each sample consisted of 10–20 specimens of aphids from zucchini or wild plants. The collected aphids were placed in 1.5-ml microtubes and stored at −80 °C. The aphids were identified to the species level under a stereo-zoom dissecting microscope using the appropriate descriptive keys [30].

### RNA extraction

RNA extraction of samples collected during the 2022–2024 survey was performed from the pooled material, grouped as follows: each zucchini and weed sample consisted of ten leaves from different plants of the same species growing nearby. The pooled plant material was ground in liquid nitrogen. Subsequently, 100 mg of powdered tissue was transferred to 1.5-ml microtubes and used for RNA extraction. In the case of previously collected zucchini plants, RNA was isolated individually from 100 mg of leaf tissue. For aphid samples, each pooled sample consisted of 10–20 individuals.

Total RNAs were extracted with the phenol/chloroform method [31] or using TRI Reagent [32]. Quality and quantity of isolated RNAs were investigated by (i) electrophoresis on agarose gel, (ii) spectrophotometrically using NanoDrop 2000 Spectrophotometer (Thermo Fisher Scientific, MA, USA) or (iii) fluorometrically using a Qubit 3 Fluorometer (Thermo Fisher Scientific), according to the manufacturer’s instructions. DNA was removed from the RNA samples using TURBO DNase (Invitrogen, MA, USA), and RNA was subsequently purified with LiCl Precipitation Solution (Invitrogen), following the manufacturer’s instructions. Prior to further analyses, the RNA samples were stored at −80 °C.

### High-throughput sequencing

Ribosomal RNA was depleted from purified RNA using RiboMinus Plant Kit for RNA-Seq (Thermo Fisher Scientific). Libraries were prepared with the VAHTS Universal V6 RNA-seq Library Prep Kit for Illumina (Vazyme Biotech, Nanjing, China), TruSeq Stranded total RNA Plant (Illumina, CA, USA) or NEBNext^®^ Ultra™ II Directional RNA Library Prep Kit for Illumina^®^ (NEB, Ipswich, USA) by weSEQ.it (Rybnik, Poland), CeGaT (Tübingen, Germany) or Genomed S.A. (Warsaw, Poland). The HTS was performed on NovaSeq 6000 (Illumina) with a 150 bp paired-end sequencing method.

The quality of raw RNA-Seq reads was assessed with FastQC [33]. Adapter-trimming, quality-trimming and filtering of the paired reads were performed with BBDuk.sh (https://sourceforge.net/projects/bbmap/). Fastq files were analysed in CLC Genomics Workbench 20.20.3 (QIAGEN, Hilden, Germany). Briefly, single FASTQ files were interleaved. For the zucchini samples, sequencing reads were first aligned to the zucchini reference genome, and host-derived reads were removed from the datasets. Subsequently, the reads from each sample were mapped separately to the National Center for Biotechnology Information (NCBI) viral RefSeq database, and the consensus sequences were recovered. Finally, out of all HTS-positive hits, 105 WMV sequences covering nearly the full-length genome were extracted and subjected to further analysis. Total number of reads, reads mapped to the reference WMV genome expressed as RPHT (reads per hundred thousand of total reads in the sample) and average coverage of the WMV genome are presented in Table S3 (available in the online Supplementary Material).

### WMV CP amplification, cloning, and Sanger sequencing

CP coding sequences of 49 WMV isolates previously collected were amplified by reverse transcription PCR (RT-PCR) using the primer pair WMVCPF/WMVCPR [34] and Transcriptor One-Step RT-PCR Kit (Roche, Basel, Switzerland), according to the manufacturer’s instructions. The presence of expected size amplification products (~977 bp in length) was checked through electrophoresis in 1% agarose gel. Subsequently, RT-PCR products were purified using NucleoSpin Gel and PCR Clean-up (Macherey-Nagel, Düren, Germany) and cloned with TOPO™ TA Cloning™ Kit for Sequencing (Invitrogen), according to the manufacturer’s instructions. Plasmid DNAs were extracted using GeneJET Plasmid Miniprep Kit (Thermo Scientific), and the presence of the insert was checked by EcoRI digestion (Thermo Scientific) according to the manufacturer’s instructions. For each isolate, three independent plasmid DNAs were bidirectionally Sanger sequenced at Genomed S.A. (Warsaw, Poland). The new CP coding sequences were assembled and edited in BioEdit [35].

### Sequence diversity of the *cp* gene in the Polish WMV isolates

Due to the lack of complete genome sequences for all isolates (especially those collected before 2022), the diversity analysis of the Polish WMV population was performed based on the CP coding region. The alignment was built based on 49 CP sequences obtained from isolates collected before 2022 and preserved in the collection, and 105 CP sequences extracted from the consensus WMV nucleotide sequences generated from HTS (Tables S1 and S3, available in the online Supplementary Material). All CP sequences were aligned together using muscle [36]. The N-terminus of CP was analysed for the presence of the DAG motif, which is involved in virus transmission by aphids, and KEKET, KEA and KET motifs diversifying WMV isolates into different phylogenetic groups.

The pairwise sequence comparisons among the Polish WMV isolates were carried out using SDT v1.3 [37]. The obtained dataset was examined for the presence of duplicate sequences. If the sequences shared 100% identity, along with a common host, region and collection date, they were excluded from the dataset (Table S2, available in the online Supplementary Material). An obtained dataset of 76 unique sequences was used in further recombination and phylogenetic analyses.

To check the variability of the Polish WMV isolates in comparison to the first described WMV isolate, the alignment of the 154 WMV CP amino acid sequences reported here and 3WMV CP partial amino acid sequences, retrieved from GenBank and obtained in 2008 (WMV-GN acc. no. FJ628395) and 2018 (D1/D2 acc. no. MH917120/MH992141), was prepared. These three sequences, due to their partial length, were not included in any of the other analyses performed here.

### Recombination and phylogenetic reconstruction

To perform the recombination analysis, 159 CP sequences of WMV were aligned using muscle [36]. Among these, 76 represented the Polish WMV population (the dataset obtained after excluding identical sequences according to the RDP4 v.4.101 recommendation [38]), and 83 retrieved from GenBank (www.https://www.ncbi.nlm.nih.gov). Recombination was analysed in RDP4 v.4.101 [38]. Seven different methods were used (RDP, GENECONV, Chimaera, MaxChi, BootScan, SiScan and 3Seq), and the occurrence of recombination was considered statistically significant if five or more methods had a *P*<0.05.

Phylogenetic analysis was performed based on the 158 nucleotide sequences of the WMV CP coding region (the recombinant isolate was excluded). As an outgroup, the SMV CP nucleotide sequence was chosen (acc. no. AJ628750.1). The sequence alignment was prepared with muscle. To perform phylogenetic reconstruction, the best-fitting substitution model was chosen with jModelTest [39]. The substitution model with the lowest Bayesian information criterion score was considered the most suitable and was, therefore, selected for further analysis. A phylogenetic maximum-likelihood (ML) tree was constructed with IQ-TREE 2 [40] and the TrN+I+G substitution model, and the confidence of the phylogeny was tested with 1,000 bootstrap replicates. The obtained tree was visualized and edited with Interactive Tree of Life (iTOL) v. 7.2.1 [41].

### Genetic variability and population structure

Firstly, the genetic variability of the Polish WMV population was investigated. Genetic diversity (π), number of haplotypes (H), haplotype diversity (Hd), number of segregating sites (S) and number of mutations (Eta) were estimated with DnaSP v6 [42]. Moreover, three different neutrality tests were performed: (i) Tajima’s *D* (where estimates of the no. of segregating sites and the mean pairwise difference between sequences are compared), (ii) Fu and Li’s *D* test (where the number of derived singleton mutations and the total number of derived nucleotide variants are compared) and (iii) Fu and Li’s *F* test (where the number of derived singleton mutations and the mean pairwise difference between sequences are compared). Sequences were grouped by (i) collection date, with division by region, (ii) collection date and (iii) region. Statistical comparisons of nucleotide diversity (π) between datasets were performed using a nonparametric bootstrap approach implemented in R, following the methodology described by Lima *et al*. [43].

Secondly, the genetic structure of the WMV global population was investigated using discriminant analysis of principal components (DAPC) [44] implemented in the Adgenet package in RStudio [45]. The analysis was performed using the dataset of WMV sequences used in phylogenetic analysis, together with identical sequences (excluded from phylogeny), in order to preserve haplotype frequencies and accurately reflect population structure. The optimal number of genetic clusters (K) was predefined with a k-means algorithm as proposed by Jombart *et al*. [44]. The number of retained principal components was determined according to the methodology described by Melo et al. 2025 [46]. Next, the relationship between DAPC-inferred clusters and phylogenetic clades was assessed by comparing cluster assignments with clade composition derived from the phylogenetic tree. The DnaSP v6 was used to calculate population genetic parameters for each phylogenetically defined clade using the dataset applied in the DAPC analysis, including genetic diversity of clades (π), number of haplotypes (H), haplotype diversity (Hd), number of segregating sites (S) and number of mutations (Eta). The neutrality Tajima’s *D*, Fu and Li’s *D* and Fu and Li’s *F* tests were performed. Moreover, the fixation index (*F*_ST_) was calculated.

Finally, episodic and pervasive selective pressure acting on individual codons in the WMV CP coding region was assessed by calculating the ratio of non-synonymous (*d*_N_) to synonymous (*d*_S_) substitution rates (ω=*d*_N_/*d*_S_). Three different approaches implemented in the Datamonkey Adaptive Evolution Server (http://www.datamonkey.org/) were used: mixed effects model of evolution (MEME), single-likelihood ancestor counting (SLAC) and fast unconstrained Bayesian approximation (FUBAR) [47,49]. The sites under positive/purifying selection were estimated under a significance value of *P*<0.05 (MEME, SLAC) or when the Bayesian posterior probabilities (BPP) were at least 0.85 (FUBAR). The recombinant isolate was excluded from this analysis.

## Results

### WMV sequence analysis

The HTS results showed that WMV was the most frequently detected virus, being found in both single and mixed infections with other plant viruses (e.g. ZYMV and CMV), with 59 and 46 sequences originating from isolates collected in the Kuyavian–Pomeranian and Greater Poland regions, respectively (Tables 1 and S1). The newly determined HTS-based CP sequences were recovered from *Aphis gossypii* (*n*=2); weeds such as *Chenopodium quinoa* (*n*=5), *Geranium pusillum* (*n*=3)*, Silene latifolia* (*n*=1), *Amaranthus* (*n*=1), *Polygonum* (*n*=1) and *Taraxacum officinale* (*n*=1); and zucchini (*C. pepo* var. *giromontiina*) (*n*=91, Tables 1 and S1). A further 49 Sanger-derived sequences of the Polish zucchini-infecting isolates collected before 2022 were obtained and included in further analysis. A total of 154 nucleotide sequences were deposited in GenBank (https://www.ncbi.nlm.nih.gov/, acc. nos. PV665942–PV666095). Nucleotide sequence analysis revealed that 78 isolates were identical. Raw FASTQ files were deposited in the Sequence Read Archive under BioProject PRJNA1237398. Detailed information regarding the origin of the Polish WMV isolates is presented in Tables 1 and S1.

**Table 1.** Detailed information about the Polish WMV population The table includes isolates, whose CP sequences were obtained in the course of this study, and three Polish isolates, collected in 2008 and 2018, whose partial CP sequences were deposited in GenBank (acc. nos. FJ628395, MH917120, MH992141).

Year	Total	Host				Region		
		Zucchini	Weeds	Aphids	Other	Kuyavian–Pomeranian	Greater Poland	No information
**2008**	1	1	–	–	–	–	–	1
**2013**	24	24	–	–	–	3	–	21
**2017**	4	4	–	–	–	–	–	4
**2018**	11	9	–	–	2	–	–	11
**2019**	11	11	–	–	–	–	–	11
**2020**	1	1	–	–	–	–	–	1
**2022**	61	50	9	2	–	40	21	–
**2023**	17	17	–	–	–	8	9	–
**2024**	27	24	3	–	–	11	16	–

The CP sequences varied in length, being either 843, 846 or 849 nt long (281, 282 and 283 aa, respectively). The slight differences in length were mostly represented by deletions of nucleotides in positions nt 7–12 or nt 13–15 in the 5′ region of the gene. One of the isolates, Fron7 (acc. no. PV666066), showed an internal deletion of 6 nt between 543 and 550 nt. The observed deletions do not show any apparent association with the geographical origin or collection date. The presence of the motifs KEKET, KEA and KET was investigated in amino acid CP sequences. The conserved motifs were found in 76 isolates, as follows: KEKET (*n*=55), KEA (*n*=11) and KET (*n*=1). An additional motif referred to as KEET was identified in nine isolates. The DAG motif was found in all the Polish WMV CP sequences.

**Fig. 2.**
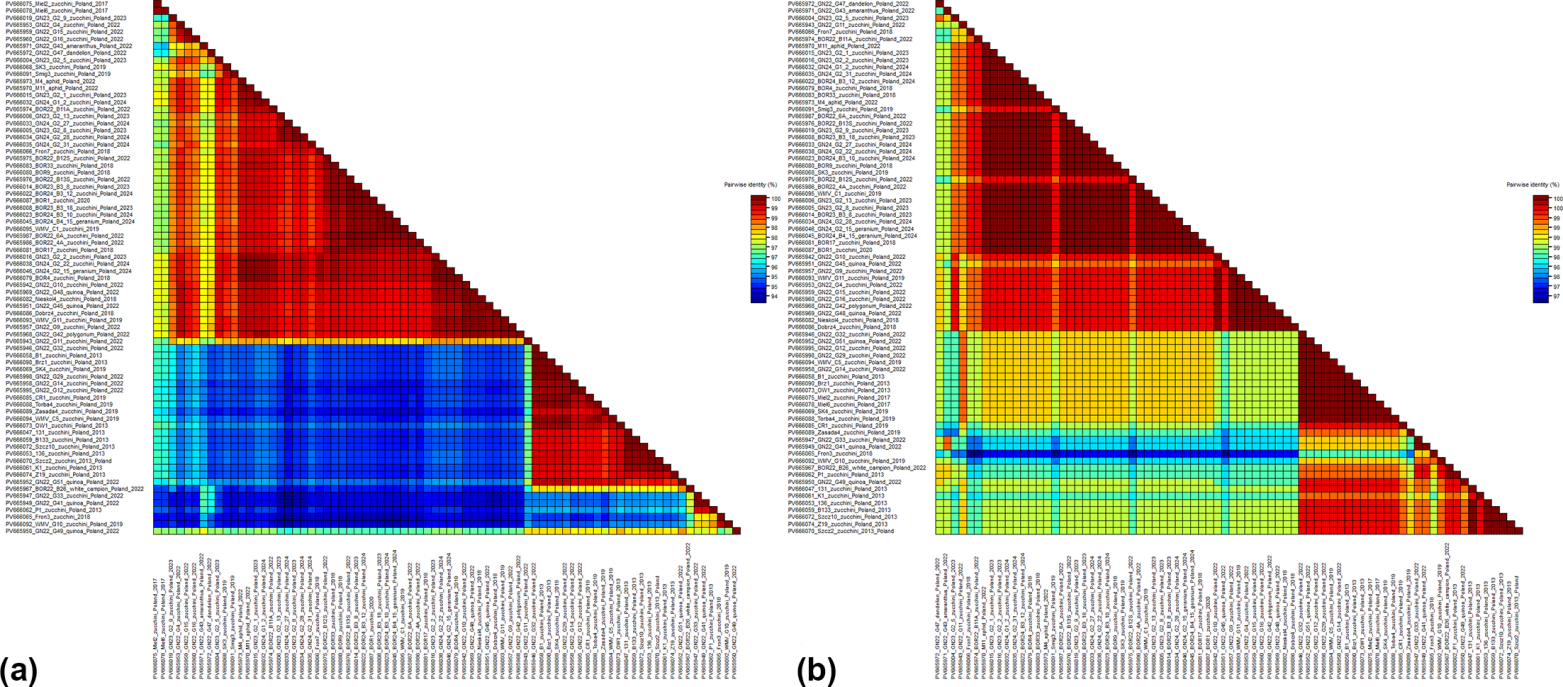
Identity matrices for (a) nucleotide and (b) amino acid CP sequences of the 76 Polish WMV isolates. Matrices were constructed in SDT v1.3.

The identity among the Polish WMV isolates ranged from 92.8 to 100% and 95.4 to 100% for nucleotide and amino acid sequences, respectively (Fig. 2a, b). Considering the sampling location of the isolates collected between 2022 and 2024, the sequences originating from the Greater Poland region exhibited 93.5% to 100% and 97.1–100% identity for nucleotide and amino acid sequences, respectively, while those from Kuyavian–Pomeranian showed 93.1 to 100% and 96.8–100% identity for nucleotide and amino acid sequences, respectively.

Among the analysed nucleotide sequences, 64% were identical and shared a common host, region and collection date (Table S2). For recombination and phylogenetic analyses, 23 representative sequences were used, fulfilling the criteria.

**Fig. 3.**
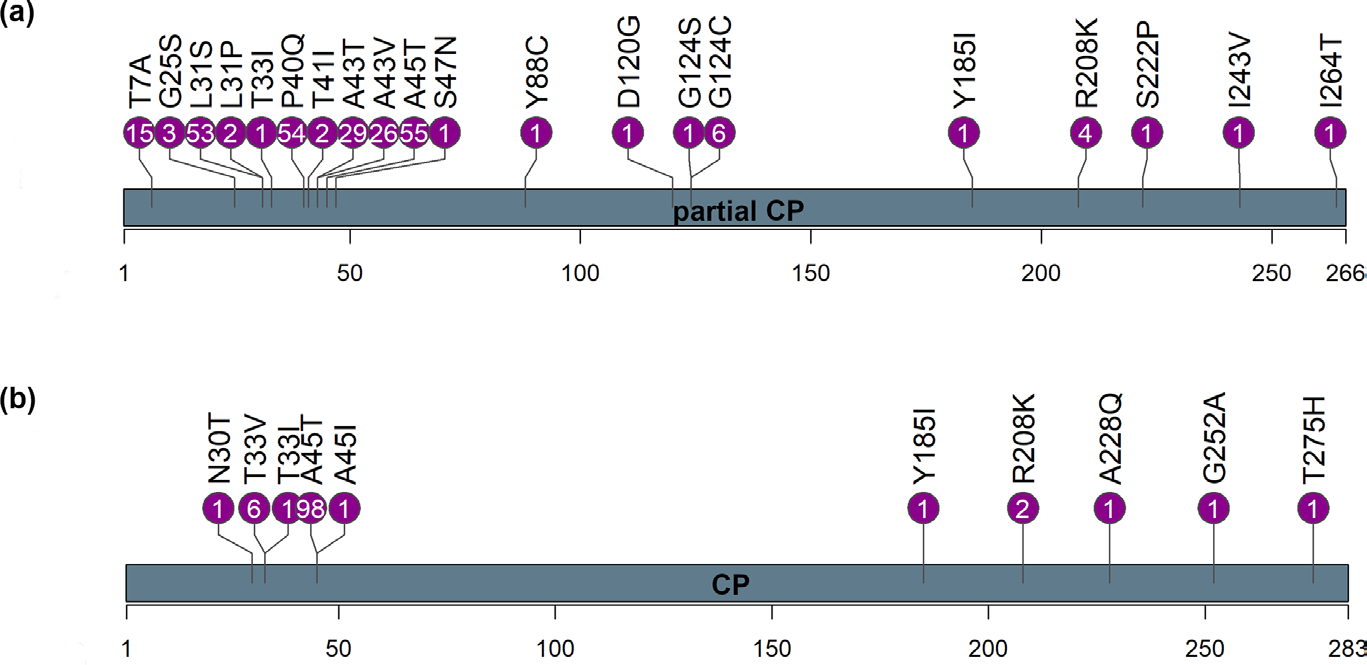
Graphical representation of (a) amino acid changes in the WMV CP fragment between 1 and 266 aa. (b) Amino acid sites being under positive/diversifying selective pressure, detected by FUBAR or MEME. For both figures, the number of sequences with an amino acid change was given in a lollipop. The figure was constructed in RStudio [64] with the trackViewer package [65].

To explore the variability of the Polish WMV isolates in comparison to the first described WMV isolates, whose partial CP coding sequence was submitted to GenBank (acc. no. FJ628395), the fragment of 266 aa was analysed. In the analysed dataset (157 sequences), 17 variable sites were detected, 9 of which were singletons, occurring in only 1 of the sequences. The highest accumulation of changes in the amino acid sequences was observed in the fragment from 1 to 50 aa, where 9 variable sites (resulting in 11 different mutations, in 2 cases in 1 aa position, 2 mutations occurred) were located (Fig. 3a). Especially interesting were changes L31S, P40Q, A43T, A43V and A45T, which occurred in 53, 54, 29, 26 and 55 out of 157 sequences, respectively (Fig. 3a). These mutations are not pervasive in isolates collected in the Greater Poland region; occurring only in 1 of the sequences (out of 46) belonging to the isolate BOR22/B26 (acc. no. PV665967), collected in 2022, originated from the *S. latifolia* growing nearby zucchini crops.

### Recombination, selection and phylogenetic reconstruction

Analysis performed with RDP4 allowed us to distinguish one potential recombinant, GN22/G16 (PV665960), belonging to the Polish WMV population (detected with five out of seven methods with *P*<0.05). Recombination breakpoints probably occurred between 452 and 807 nt of the CP coding region, with putative parental sequences being a French isolate EM170143 (acc. no. PP505905) infecting melon (*Cucumis melo*), detected in 2017, and a South Korean isolate (acc. no. MW483119) infecting butternut squash (*Cucurbita moschata*). Isolate GN22/G16 was collected in 2022 from zucchini growing in the Kuyavian–Pomeranian region in Poland.

**Fig. 4.**
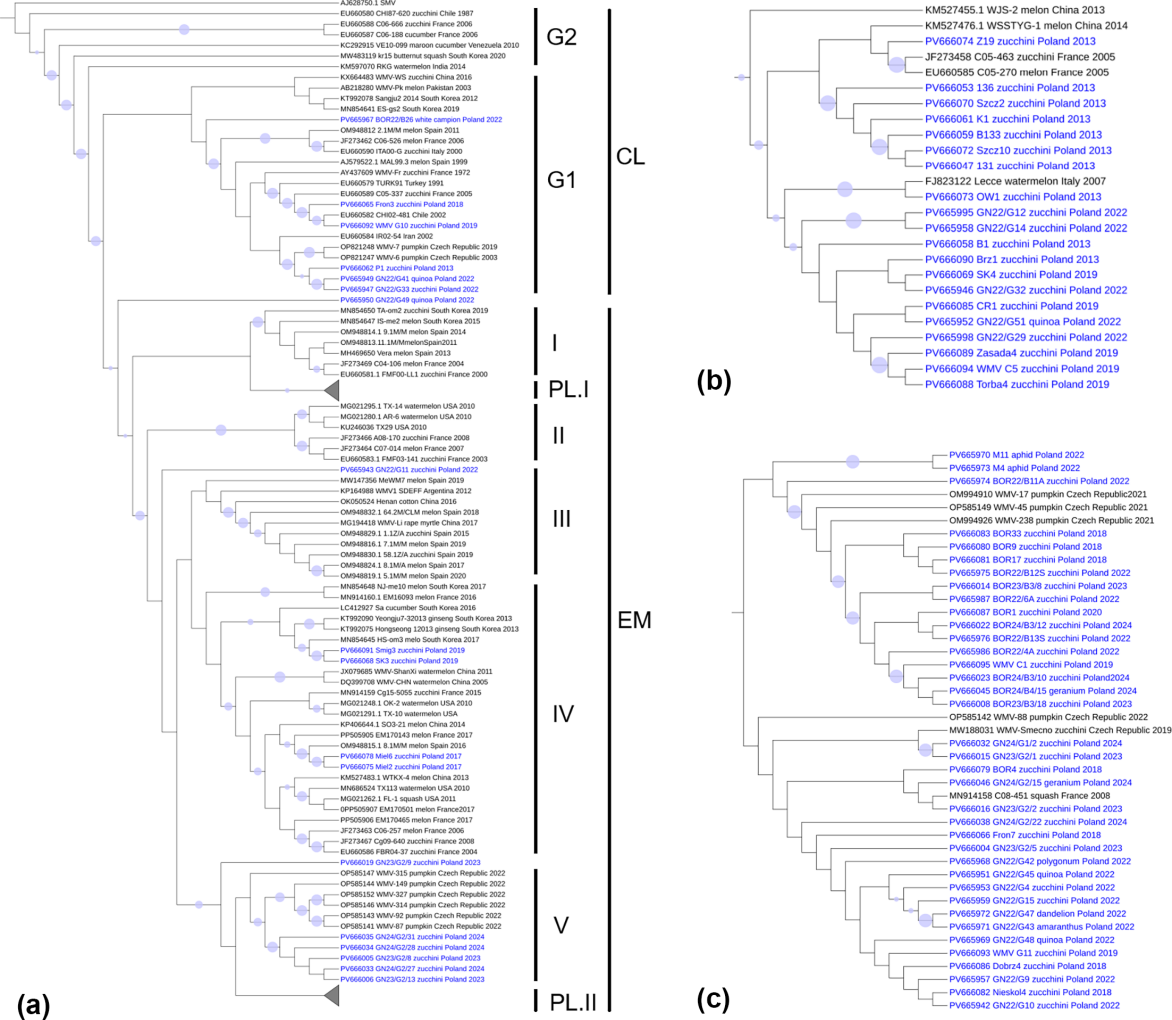
(a) Phylogenetic ML tree constructed based on 158 nt WMV CP sequences. (b) Expansion of the subgroup PL.I. (c) Expansion of the subgroup PL.II. Circles on branches represent bootstrap support >80%. For each isolate accession number, isolate name, host, origin and collection year are given. Polish WMV isolates are marked in blue. The tree was constructed in IQ-TREE [40], with the TrN+G+I model. Visualization and editing were performed in iTOL.

The ML tree was constructed based on 158 complete coding sequences of the WMV *cp* gene, 75 of which originated from the Polish WMV isolates and 83 were retrieved from GenBank, belonging to isolates collected from various locations and host plants, with the prevalence of those originating from zucchini (Fig. 4). The analysis revealed two main clades. The first clade, previously described as the classical lineage, was further divided by us into groups G1 and G2, following earlier literature reports [28,50]. This clade includes mostly well-known, widely described WMV isolates. G2 consists of six isolates: CH87-620 (1987, Chile), VE10-099 (2010, Venezuela), kr15 (2020, South Korea), RGK (2014, India) and two French isolates, C06-666 and C06-188, collected in 2006 (Fig. 4a).

Group G1 contains both basal classical isolates, collected between 1972 (WMF-Fr) and 2006, as well as more recently identified ones. Notably, six Polish isolates cluster within this group: BOR22/B26, Fron3, WMV G10, P1, GN22/G41 and GN22/G33 (Fig. 4a). These isolates are inconsistent in terms of both collection date and host plant. Four were obtained from zucchini, while the remaining two were isolated from weeds (quinoa and white campion). These two, together with two South Korean isolates (acc. no. KT992078, MN854641, originating from ginseng), are the only non-cucurbitaceous samples in the group (4 out of 24).

The second clade comprises predominantly isolates detected within the last decade and is commonly referred to as the emerging lineage. Based on the ML tree, seven distinct subgroups were clearly defined: I, PL.I, II, III, IV, V and PL.II (Fig. 4a). Subgroup I consists of South Korean, Spanish and French isolates originating from zucchini and melon. Subgroup II includes six cucurbitaceous-origin isolates from the USA and France. Subgroup III comprises Spanish isolates collected between 2015 and 2020, two Chinese isolates and one from Argentina. Subgroup IV includes isolates from South Korea, China and the USA, along with European isolates from France, Spain and four from Poland (Smig3, SK3, Miel6 and Miel2), collected in 2019 and 2017, respectively. Subgroup V contains isolates from the Czech Republic (*n*=6) and Poland (*n*=5), collected in two different years (2023 and 2024), but originating from the same region of the country.

The majority of the Polish WMV isolates form two distinct subgroups, named PL.I and PL.II due to their prevalence (Fig. 4b, c, respectively). PL.I mainly consists of isolates obtained before 2022, particularly in 2013. Only five isolates in this subgroup were collected during the 2022 zucchini crop survey in the Kuyavian–Pomeranian region. Non-Polish isolates within PL.I subgroup include the Italian isolate Lecce, two French isolates (C05-463, C05-270) and two Chinese isolates, WSSTYG-1 and WJS-2, isolated from melon. In contrast, PL.II predominantly contains Polish isolates collected after 2021. Within this group, a clear clustering by collection region (Greater Poland or Kuyavian–Pomeranian) can be observed. Similar to subgroup V, PL.II also includes WMV isolates from the Czech Republic and one isolate collected in France.

### Genetic variability and population structure

**Fig. 5.**
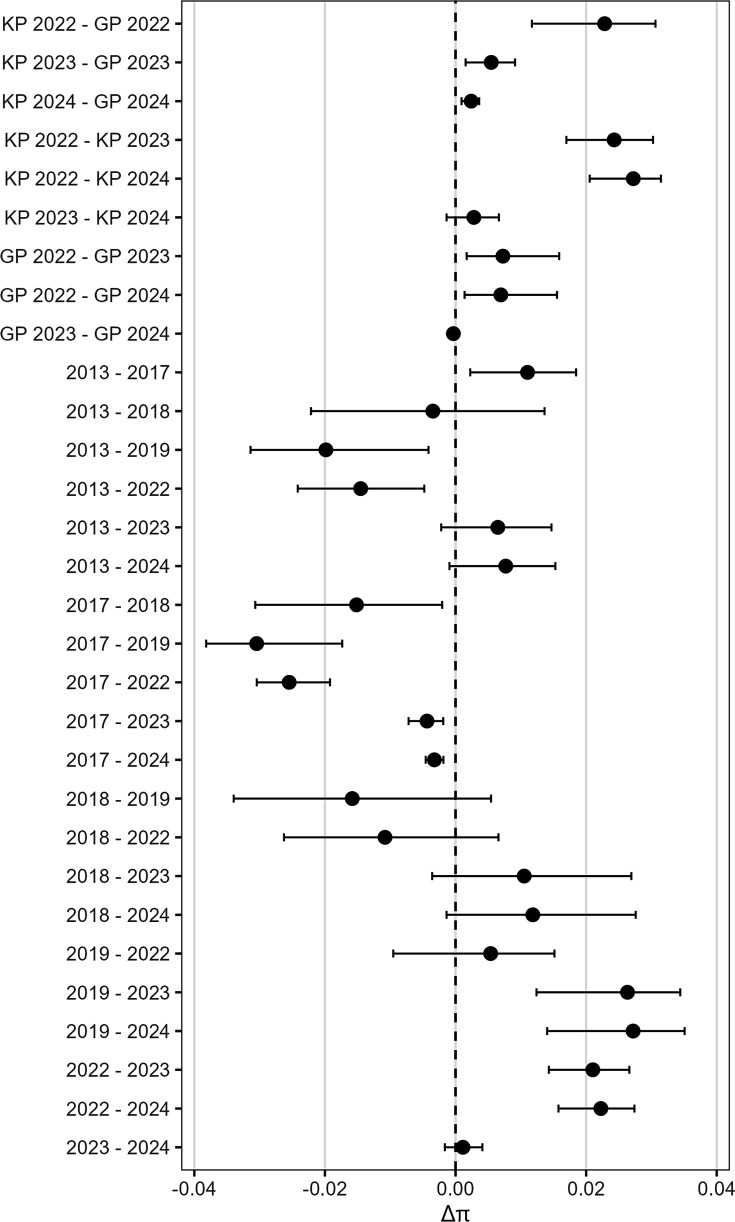
Statistical significance of nucleotide diversity (π) calculated for the CP coding sequences of the Polish WMV population. Ninety-five percent bootstrap confidence intervals (CIs) for differences in π values were estimated using 15,000 nonparametric simulations in RStudio. Intervals including zero indicated no significant differences between the means. Graphical visualization was generated in RStudio [64] using the ggplot2 package [66].

Analysis of a particular WMV subpopulations distinguished by origin (Kuyavian–Pomeranian or Greater Poland regions) and collection date (2022–2024) revealed relatively low nucleotide diversity, especially in the case of isolates collected in Greater Poland, where the π was 0.0078, 0.0003 and 0.0006 for 2022, 2023 and 2024, respectively (Table 2). For both regions, the most diverse subpopulations were those grouping isolates collected in 2022 (0.0307 and 0.0078 for Kuyavian–Pomeranian and Greater Poland, respectively). Correspondingly, subpopulations of the two regions differed in terms of the number of segregation sites (S), and only for the Kuyavian–Pomeranian subpopulation from 2022, the total number of mutations (Eta) was higher than S (Table 2). The largest numbers of S display the subpopulations of Kuyavian–Pomeranian and Greater Poland isolates collected in 2022 (68 and 50, respectively). The Tajima *D*, Fu and Li’s *D* and Fu and Li’s *F* tests were statistically significant only for subpopulations collected in 2022, with the positive values for subpopulation originating from Kuyavian–Pomeranian and negative for those from Greater Poland. Considering WMV subpopulations without division for origin, the lowest nucleotide diversity was exhibited in the subpopulation collected in 2017, and the highest collected in 2019. All Tajima’s *D* test results were nonsignificant, whereas Fu and Li’s *D*, and Fu and Li’s *F* test results were statistically significant only for isolates collected in 2022. To further assess temporal and spatial variation in nucleotide diversity, statistical comparisons of π between subpopulations were performed using a nonparametric bootstrap approach (Fig. 5). Statistical comparisons of π confirmed significant temporal variation among WMV populations (Fig. 5). WMV subpopulations collected in 2022 exhibited significantly higher π compared to those from 2023 and 2024, as indicated by non-overlapping 95% confidence intervals. A similar pattern was observed for isolates collected in 2019, which also showed an increased level of genetic diversity relative to those collected in 2023 and 2024. Subpopulations collected in those years exhibited reduced genetic diversity compared to earlier years. A significant difference between regions was detected only in 2022, with the Kuyavian–Pomeranian subpopulation showing higher π than the population originating in Greater Poland, whereas no significant regional differences were observed in 2023 and 2024 (Fig. 5).

**Table 2.** Genetic diversity of the Polish WMV population

Region	Collection date	N	H	Hd	π	S	Eta	Tajima *D*	Fu and Li’s *D*	Fu and Li’s *F*
**Kuyavian–Pomeranian**	2022	40	14	0.805	0.0307	68	70	2.2161*	1.8474**	2.3521**
	2023	8	6	0.893	0.0065	19	19	−1.3203^ns^	−1.2519^ns^	−1.4100^ns^
	2024	11	5	0.764	0.0032	9	9	−0.4725^ns^	−1.1013^ns^	−1.0652^ns^
**Greater Poland**	2022	21	6	0.738	0.0078	50	50	−2.1162*	−3.3753**	−3.4957**
	2023	9	2	0.222	0.0003	1	1	−1.0882^ns^	−1.1899^ns^	−1.2829^ns^
	2024	16	2	0.500	0.0006	1	1	1.3090^ns^	0.6883^ns^	0.9651^ns^
**Total**	2013	24	7	0.728	0.0117	41	42	−0.3935^ns^	1.2841^ns^	0.8925^ns^
	2017	4	2	0.500	0.0006	1	1	−0.6124^ns^	−0.6124^ns^	−0.4787^ns^
	2018	9	8	0.972	0.0162	55	55	−1.6870^ns^	−1.8859^ns^	−2.0638^ns^
	2019	11	9	0.964	0.0337	77	79	0.3699^ns^	−0.3474^ns^	−0.1850^ns^
	2020	1	–	–	–	–	–	–	–	–
	2022	61	20	0.887	0.0263	74	76	1.3588^ns^	1.8721**	2.0049**
	2023	17	8	0.772	0.0052	23	23	−1.4413^ns^	−1.9312^ns^	−2.0718^ns^
	2024	27	7	0.792	0.0038	13	13	−0.1251^ns^	−1.0643^ns^	−0.9081^ns^

*P<0.05; **P<0.02; ns, non-significant.

N, number of sequences; H, number of haplotypes; Hd, haplotype diversity; π, nucleotide diversity; S, segregating sites; Eta, total number of mutations.

**Fig. 6.**
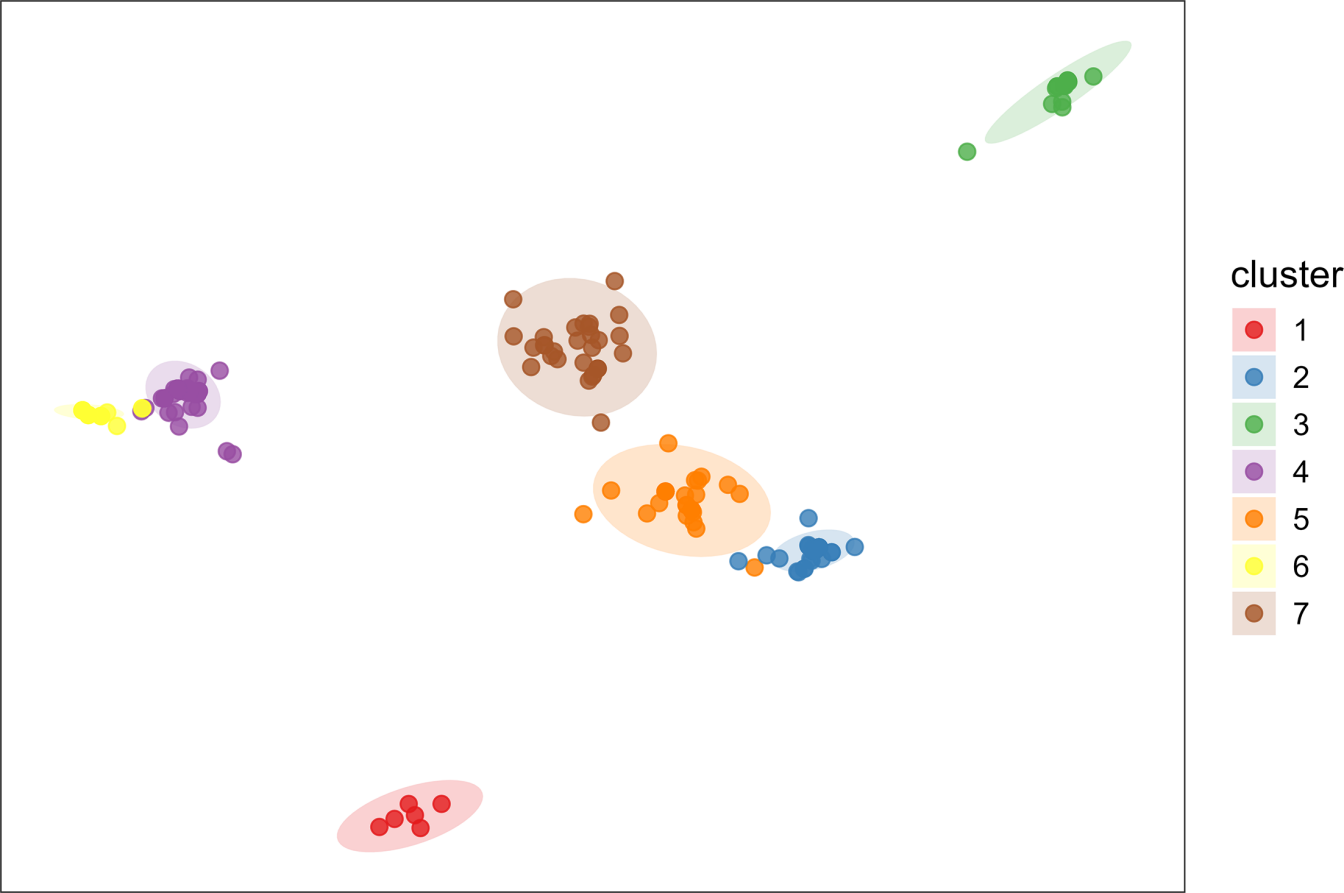
Scatter plot presenting population structure of WMV in accordance with DAPC. Graphical visualization was generated in RStudio [64] using the ggplot2 package [66].

The DAPC analysis indicated that the WMV population is structured into seven genetic clusters, with most of them showing clear separation in the discriminant space (Fig. 6). The analysis revealed strong correspondence between DAPC clusters and phylogenetically defined clades. Clusters 5 and 1 corresponded to clades G1 and G2, respectively, reflecting clear genetic differentiation, although they were positioned relatively close to other clusters in the discriminant space. Some clusters grouped closely related lineages. In particular, isolates from clades I and PL.I were assigned to the same cluster (cluster 2) as those from clades II and III (cluster 3), indicating low genetic divergence among these groups. Notably, isolates belonging to phylogenetic subgroup PL.II were divided into two DAPC clusters: one consisting exclusively of PL.II isolates (cluster 6) and another grouping PL.II together with clade V (cluster 4), indicating internal genetic structure within this clade and a close genetic relationship with clade V. Although a similar subdivision can be observed in the phylogenetic tree, the assignment of isolates to these subgroups is not fully consistent between the two approaches. Clade IV formed a largely distinct cluster (cluster 7), with only minor overlap with other groups.

Population genetics analyses revealed that although subpopulations of WMV differed in the number of unique gene variants (H), all exhibited high haplotype diversity (Hd ranging from 0.840 to 1.000, Table 3). Overall, low nucleotide diversity was observed across the WMV population, with the highest values for clade G2 (π=0.0406) and the lowest for clade PL.II (π=0.0048), which corresponded with the number of segregating sites (S) and the total number of mutations (Eta) (Table 3). Neutrality tests, including Tajima’s *D*, Fu and Li’s *D* and Fu and Li’s *F*, were applied to assess whether natural selection or genetic drift has influenced viral evolution (Table 3). All clades exhibited negative values for these statistics, suggesting an excess of rare alleles. However, only clades PL.I and PL.II showed statistically significant deviations from neutrality, indicating a recent demographic expansion or positive selection acting on this population. *F_ST_* values ranged from 0.2867 (clade V vs. PL.II) to 0.9013 (clade PL.I vs. PL.II) (Table 4). These relatively high levels of differentiation suggest limited or no gene flow between subpopulations and support the existence of distinct genetic structure among WMV clades.

**Table 3.** Genetic diversity of the WMV population

Clade	N	H	Hd	π	S	Eta	Tajima *D*	Fu and Li’s *D*	Fu and Li’s *F*
**G1**	26	20	0.960	0.0213	107	114	−1.5685^ns^	−2.1028^ns^	−2.2739^ns^
**G2**	6	6	1	0.0406	82	87	−0.6611^ns^	−0.5476^ns^	−0.6281^ns^
**I**	7	7	1	0.0071	19	19	−1.2675^ns^	−1.2450^ns^	−1.3776^ns^
**II**	6	6	1	0.0160	32	32	−0.1877^ns^	−0.4286^ns^	−0.4125^ns^
**III**	10	10	1	0.0224	72	74	−1.3493^ns^	−1.2422^ns^	−1.4339^ns^
**IV**	28	25	0.989	0.0195	114	118	−1.7788^ns^	−2.4497^ns^	−2.6320^ns^
**V**	13	10	0.923	0.0050	16	16	−0.7748^ns^	−1.1994^ns^	−1.2410^ns^
**PL.I**	42	15	0.840	0.0054	36	36	−1.5715ns	−2.6793*	−2.7241*
**PL.II**	95	26	0.856	0.0048	48	48	−1.8160^ns^	−3.7482**	−3.5626**

*P<0.05; **P<0.02; ns, non-significant.

N, number of sequences; H, number of haplotypes; Hd, haplotype diversity; π, nucleotide diversity; S, segregating sites; Eta, total number of mutations. Particular populations were divided based on the topology of the phylogenetic tree (Fig. 4).

**Table 4.** Genetic differentiation of the WMV population based on Wright’s *F_ST_* index Populations were divided based on the topology of the phylogenetic tree (Fig. 4).

Clade	G1	G2	I	II	III	IV	V	PL.I	Pl.II
**G1**	–	0.4617	0.7279	0.7251	0.6703	0.6460	0.7722	0.7084	0.7683
**G2**	–	–	0.5867	0.6570	0.6004	0.5541	0.6564	0.5590	0.6557
**I**	–	–	–	0.7911	0.7382	0.7366	0.8818	0.8089	0.8833
**II**	–	–	–	–	0.6164	0.7236	0.8346	0.8172	0.8385
**III**	–	–	–	–	–	0.5982	0.7447	0.7692	0.7460
**IV**	–	–	–	–	–	–	0.3835	0.7553	0.3653
**V**	–	–	–	–	–	–	–	0.8998	0.2867
**PL.I**	–	–	–	–	–	–	–	–	0.9013

The likelihood ratio test has shown that episodic diversifying selection has acted on seven sites in the analysed dataset (codons: 30, 33, 185, 208, 228, 252 and 275, detected by MEME, *P*<0.05). Moreover, one site under pervasive positive/diversifying selection was detected by FUBAR (codon 45, BPP<0.85). Results were presented in Fig. 3(b).

## Discussion

Cucurbits are one of the major vegetables cultivated worldwide, exposed to various viral infections, with the prevalence of those belonging to the *Potyvirus* family, including WMV [25]. WMV was first detected in Poland in 2008 [17]. Since then, only a few reports regarding virus occurrence have been published [18,20,51]. The identification, classification and analysis of plant viruses have become increasingly accessible thanks to HTS and the continuous development of advanced bioinformatic tools. Using HTS allowed sequencing over 1,800 zucchini and weed samples collected during surveys in the Kuyavian–Pomeranian and Greater Poland regions of Poland.

In this study, the diversity and structure of the Polish WMV population were investigated based on the 154 complete coding sequences of CP. The results indicate that the Polish WMV population exhibits a sequence diversity ranging from 92.8 to 100% at the nucleotide level and from 95.4 to 100% at the amino acid level. This diversity appears to be only minimally influenced by the geographic region in which the isolates were collected. Within the population, multiple identical sequences were found, not only within the isolated collections from the same region but also within those separated by time (detected in different years) and distance. The high similarity observed between allopatric viral populations exposed to similar environments can be explained by strong purifying selection, which removes deleterious mutations and favours genetic stability [52].

The analysed sequences differed in length, and this variation was associated with the presence or absence of the KEKET, KEA and KET motifs. These motifs, located between positions 3 and 7 aa at the N-terminus of the CP, were previously identified in representatives of different phylogenetic groups of WMV (G3 – emerging isolates and the classical G1 and G2 groups) [24,53]. Moreover, the KEKET and KEA motifs are suspected to play a role in symptom modulation [53]. Interestingly, in the case of nine Polish isolates, the unique motif KEET at the same position was found. This type of amino acid sequence has not been described in the literature before. In all of the analysed sequences, a highly conserved motif DAG was found. This motif directly interacts with a PTK motif (or its functionally equivalent motif/s) located in the C-terminus of HC-Pro and is involved in virus transmission through aphids [54,55]. In the Polish WMV population, one recombinant was found; the potential recombination breakpoints were located between 452 and 807 nt of the *cp*. Mutations and recombination are the main forces driving virus evolution. Previous research indicated that recombination is pervasive for WMV and multiple recombination events in different parts of the virus genome, including the *cp* gene, were identified [28,56,58]. Abdalla and Ali [24] identified seven distinct recombination breakpoints within the *cp* gene; however, none of them overlapped with those detected in our study. In WMV, the prevalence of negative (purifying) selection acting on specific codons was confirmed. Only eight amino acid sites were found to be under positive (diversifying) selection, and none of them were identified consistently by more than one method. These results confirmed those previously published for the WMV [24,59]. Multiple analyses concerning WMV molecular evolution and phylodynamics have been performed, with the most global up-to-date presented by Rabadán and Gómez [29]. The authors confirmed WMV separation into two well-defined clades (classical and emergent) and the polyphyletic origin of the WMV population. Phylogenetic relationships between the Polish WMV isolates and those distributed worldwide were also assessed, where classical G1 and G2 and an emerging phylogenetic group (EM) were distinguished. The analysis showed a high level of population differentiation and low genetic flow between WMV subpopulations. Interestingly, the Polish isolates were grouped in both main phylogenetic clades: CL and the EM. Despite the fact that historically the CL clade (G1 and G2) subgroup was reserved for former isolates (detected mainly before 2000), the classical isolates have also been identified in other countries; however, their prevalence in local virus populations is diverse. In our studies, among 75 Polish WMV isolates, only six were grouped into G1, and none of them into the G2 subgroup. Rabadán and Gómez [29] sequenced and analysed 22 Spanish WMV isolates, and among them, only 2 were grouped in the classical group. It contrasts with the analysis performed by Hajizadeh *et al*. [60], where the previously sequenced Spanish WMV isolates, collected before 2000 [61], were exclusively grouped into the CL clade. Among the Turkish WMV isolates [62], 11 out of 19 isolates were classified into the G1 group. Abdalla and Ali [24] showed that all of the USA isolated collected between 2007 and 2011 in different US states belong to the EM isolates and were divided into different distinct subgroups. The tree topology suggests that, to some extent, the country of origin may influence isolate clustering, whereas the host plant appears to have a lesser effect. However, this hypothesis should be further tested using a more diverse set of isolates with regard to host plant species. Grouping of WMV isolates in distantly related phylogenetic groups can suggest various introduction events [50]. On the other hand, the presence of two distinct subgroups containing the Polish WMV isolates may reflect a unique introduction event followed by the subsequent diversification of the viral population. The close phylogenetic relationship between Polish and Czech isolates [63] may suggest their common origin. It has been proposed that WMV isolates may spread to other geographical regions through the movement of propagated plant material and subsequently evolve via genetic drift [60].

The DAPC analysis revealed a well-defined population structure largely consistent with phylogenetic clades, with partial overlap among closely related lineages. These findings suggest that while phylogenetic clades represent distinct evolutionary units, some of them form higher-level genetic groupings detectable by model-free approaches such as DAPC.

Temporal variation in nucleotide diversity, with higher values observed in earlier sampling years, suggests dynamic changes in WMV population structure over time. Similarly, in the case of the Polish WMV population, as well as within the subpopulations defined by phylogenetic analysis, genetic drift appears to be the predominant evolutionary force shaping viral diversity. The information obtained in this study significantly expanded our knowledge regarding WMV variability in Poland and provides a comprehensive analysis of its population structure, which could be beneficial in epidemiological studies, as well as virus management.

## Supplementary material

10.1099/jgv.0.002273 Supplementary Material 1.

## Abbreviations

BPP: Bayesian posterior probabilities
CL: classical
CMV: cucumber mosaic virus
DAPC: discriminant analysis of principal components
EM: emergent
FUBAR: fast unconstrained Bayesian approximation
HTS: high-throughput sequencing
iTOL: Interactive Tree of Life
MEME: mixed effects model of evolution
ML: maximum likelihood
Mt: megatonnes
nt: nucleotides
PRSV: papaya ringspot virus
SLAC: single-likelihood ancestor counting
SMV: soybean mosaic virus
WMV: watermelon mosaic virus
ZYMV: zucchini yellow mosaic virus
