# Supplementary material for: Variability and population structure of watermelon mosaic virus in zucchini crops in Poland
Source: J Gen Virol. 2026 Jul 3;107(7):002273. doi: 10.1099/jgv.0.002273 (PMC13378793; doi:10.1099/jgv.0.002273)
Supplement: Supplementary Material 1. [file jgv-107-02273-s001.pdf]

Table S1. Polish WMV isolates whose CP sequences were obtained in the course of this study; KP – Kuyavian-Pomeranian region, GP – Greater Poland region.

Accession no.	Isolate	Host	Collection date	Region	Sequencing method
PV666047	131	*Cucurbita pepo var. giromontiina*	2013	Poland	Sanger
PV666048	132	*Cucurbita pepo var. giromontiina*	2013	Poland	Sanger
PV666049	133	*Cucurbita pepo var. giromontiina*	2013	Poland	Sanger
PV666050	134	*Cucurbita pepo var. giromontiina*	2013	Poland	Sanger
PV666051	134z	*Cucurbita pepo var. giromontiina*	2013	Poland	Sanger
PV666052	135	*Cucurbita pepo var. giromontiina*	2013	Poland	Sanger
PV666053	136	*Cucurbita pepo var. giromontiina*	2013	Poland	Sanger
PV666054	137z	*Cucurbita pepo var. giromontiina*	2013	Poland	Sanger
PV666055	138	*Cucurbita pepo var. giromontiina*	2013	Poland	Sanger
PV666056	139	*Cucurbita pepo var. giromontiina*	2013	Poland	Sanger
PV666057	1310	*Cucurbita pepo var. giromontiina*	2013	Poland	Sanger
PV666058	B1	*Cucurbita pepo var. giromontiina*	2013	Poland	Sanger
PV666059	B133	*Cucurbita pepo var. giromontiina*	2013	Poland	Sanger
PV666060	B134	*Cucurbita pepo var. giromontiina*	2013	Poland	Sanger
PV666061	K1	*Cucurbita pepo var. giromontiina*	2013	Poland	Sanger
PV666062	P1	*Cucurbita pepo var. giromontiina*	2013	Poland	Sanger
PV666063	P5	*Cucurbita pepo var. giromontiina*	2013	Poland	Sanger
PV666064	P6	*Cucurbita pepo var. giromontiina*	2013	Poland	Sanger
PV666065	Fron3	*Cucurbita pepo var. giromontiina*	2018	Poland	Sanger
PV666066	Fron7	*Cucurbita pepo var. giromontiina*	2018	Poland	Sanger
PV666067	SK1	*Cucurbita pepo var. giromontiina*	2019	Poland	Sanger

Accession no.	Isolate	Host	Collection date	Region	Sequencing method
**PV666068**	SK3	*Cucurbita pepo var. giromontiina*	2019	Poland	Sanger
**PV666069**	SK4	*Cucurbita pepo var. giromontiina*	2019	Poland	Sanger
**PV666070**	Szcz2	*Cucurbita pepo var. giromontiina*	2013	Poland	Sanger
**PV666071**	Szcz7	*Cucurbita pepo var. giromontiina*	2013	Poland	Sanger
**PV666072**	Szcz10	*Cucurbita pepo var. giromontiina*	2013	Poland	Sanger
**PV666073**	OW1	*Cucurbita pepo var. giromontiina*	2013	Poland	Sanger
**PV666074**	Z19	*Cucurbita pepo var. giromontiina*	2013	Poland	Sanger
**PV666075**	Miel2	*Cucurbita pepo var. giromontiina*	2017	Poland	Sanger
**PV666076**	Miel3	*Cucurbita pepo var. giromontiina*	2017	Poland	Sanger
**PV666077**	Miel5	*Cucurbita pepo var. giromontiina*	2017	Poland	Sanger
**PV666078**	Miel6	*Cucurbita pepo var. giromontiina*	2017	Poland	Sanger
**PV666079**	BOR4	*Cucurbita pepo var. giromontiina*	2018	Poland	Sanger
**PV666080**	BOR9	*Cucurbita pepo var. giromontiina*	2018	Poland	Sanger
**PV666081**	BOR17	*Cucurbita pepo var. giromontiina*	2018	Poland	Sanger
**PV666082**	Nieskol4	*Cucurbita pepo var. giromontiina*	2018	Poland	Sanger
**PV666083**	BOR33	*Cucurbita pepo var. giromontiina*	2018	Poland	Sanger
**PV666084**	BOR45	*Cucurbita pepo var. giromontiina*	2018	Poland	Sanger
**PV666085**	CR1	*Cucurbita pepo var. giromontiina*	2019	Poland	Sanger
**PV666086**	Dobrz4	*Cucurbita pepo var. giromontiina*	2018	Poland	Sanger
**PV666087**	BOR1	*Cucurbita pepo var. giromontiina*	2020	Poland	Sanger
**PV666088**	Torba4	*Cucurbita pepo var. giromontiina*	2019	Poland	Sanger
**PV666089**	Zasada4	*Cucurbita pepo var. giromontiina*	2019	Poland	Sanger
**PV666090**	Brz1	*Cucurbita pepo var. giromontiina*	2013	Poland	Sanger

Accession no.	Isolate	Host	Collection date	Region	Sequencing method
**PV666091**	Smig3	*Cucurbita pepo var. giromontiina*	2019	Poland	Sanger
**PV666092**	WMV_G10	*Cucurbita pepo var. giromontiina*	2019	Poland	Sanger
**PV666093**	WMV_G11	*Cucurbita pepo var. giromontiina*	2019	Poland	Sanger
**PV666094**	WMV_C5	*Cucurbita pepo var. giromontiina*	2019	Poland	Sanger
**PV666095**	WMV_C1	*Cucurbita pepo var. giromontiina*	2019	Poland	Sanger
**PV665942**	GN22/G10	*Cucurbita pepo var. giromontiina*	2022	KP, Poland	HTS
**PV665943**	GN22/G11	*Cucurbita pepo var. giromontiina*	2022	KP, Poland	HTS
**PV665944**	GN22/G17	*Cucurbita pepo var. giromontiina*	2022	KP, Poland	HTS
**PV665945**	GN22/G24	*Cucurbita pepo var. giromontiina*	2022	KP, Poland	HTS
**PV665946**	GN22/G32	*Cucurbita pepo var. giromontiina*	2022	KP, Poland	HTS
**PV665947**	GN22/G33	*Cucurbita pepo var. giromontiina*	2022	KP, Poland	HTS
**PV665948**	GN22/G34	*Cucurbita pepo var. giromontiina*	2022	KP, Poland	HTS
**PV665949**	GN22/G41	*Chenopodium quinoa*	2022	KP, Poland	HTS
**PV665950**	GN22/G49	*Chenopodium quinoa*	2022	KP, Poland	HTS
**PV665951**	GN22/G45	*Chenopodium quinoa*	2022	KP, Poland	HTS
**PV665952**	GN22/G51	*Chenopodium quinoa*	2022	KP, Poland	HTS
**PV665953**	GN22/G4	*Cucurbita pepo var. giromontiina*	2022	KP, Poland	HTS
**PV665954**	GN22/G5	*Cucurbita pepo var. giromontiina*	2022	KP, Poland	HTS
**PV665955**	GN22/G6	*Cucurbita pepo var. giromontiina*	2022	KP, Poland	HTS
**PV665956**	GN22/G7	*Cucurbita pepo var. giromontiina*	2022	KP, Poland	HTS
**PV665957**	GN22/G9	*Cucurbita pepo var. giromontiina*	2022	KP, Poland	HTS
**PV665958**	GN22/G14	*Cucurbita pepo var. giromontiina*	2022	KP, Poland	HTS
**PV665959**	GN22/G15	*Cucurbita pepo var. giromontiina*	2022	KP, Poland	HTS

Accession no.	Isolate	Host	Collection date	Region	Sequencing method
**PV665960**	GN22/G16	*Cucurbita pepo var. giromontiina*	2022	KP, Poland	HTS
**PV665961**	GN22/G20	*Cucurbita pepo var. giromontiina*	2022	KP, Poland	HTS
**PV665962**	GN22/G22	*Cucurbita pepo var. giromontiina*	2022	KP, Poland	HTS
**PV665963**	GN22/G23	*Cucurbita pepo var. giromontiina*	2022	KP, Poland	HTS
**PV665964**	GN22/G25	*Cucurbita pepo var. giromontiina*	2022	KP, Poland	HTS
**PV665965**	GN22/G26	*Cucurbita pepo var. giromontiina*	2022	KP, Poland	HTS
**PV665966**	GN22/G27	*Cucurbita pepo var. giromontiina*	2022	KP, Poland	HTS
**PV665967**	BOR22/B26	*Silene latifolia*	2022	GP, Poland	HTS
**PV665968**	GN22/G42	*Polygonum*	2022	KP, Poland	HTS
**PV665969**	GN22/G48	*Chenopodium quinoa*	2022	KP, Poland	HTS
**PV665970**	M11	*Aphis gossypi*	2022	GP, Poland	HTS
**PV665971**	GN22/G43	*Amaranthus*	2022	KP, Poland	HTS
**PV665972**	GN22/G47	*Taraxacum officinale*	2022	KP, Poland	HTS
**PV665973**	M4	*Aphis gossypi*	2022	GP, Poland	HTS
**PV665974**	BOR22/B11A	*Cucurbita pepo var. giromontiina*	2022	GP, Poland	HTS
**PV665975**	BOR22/B12S	*Cucurbita pepo var. giromontiina*	2022	GP, Poland	HTS
**PV665976**	BOR22/B13S	*Cucurbita pepo var. giromontiina*	2022	GP, Poland	HTS
**PV665977**	BOR22/B14A	*Cucurbita pepo var. giromontiina*	2022	GP, Poland	HTS
**PV665978**	BOR22/B16A	*Cucurbita pepo var. giromontiina*	2022	GP, Poland	HTS
**PV665979**	BOR22/B17A	*Cucurbita pepo var. giromontiina*	2022	GP, Poland	HTS
**PV665980**	GN22/G1	*Cucurbita pepo var. giromontiina*	2022	KP, Poland	HTS
**PV665981**	GN22/G2	*Cucurbita pepo var. giromontiina*	2022	KP, Poland	HTS
**PV665982**	GN22/G3	*Cucurbita pepo var. giromontiina*	2022	KP, Poland	HTS

Accession no.	Isolate	Host	Collection date	Region	Sequencing method
**PV665983**	BOR22/B10A	*Cucurbita pepo var. giromontiina*	2022	GP, Poland	HTS
**PV665984**	BOR22/B36	*Cucurbita pepo var. giromontiina*	2022	GP, Poland	HTS
**PV665985**	BOR22/2S	*Cucurbita pepo var. giromontiina*	2022	GP, Poland	HTS
**PV665986**	BOR22/4A	*Cucurbita pepo var. giromontiina*	2022	GP, Poland	HTS
**PV665987**	BOR22/6A	*Cucurbita pepo var. giromontiina*	2022	GP, Poland	HTS
**PV665988**	BOR22/7A	*Cucurbita pepo var. giromontiina*	2022	GP, Poland	HTS
**PV665989**	BOR22/8S	*Cucurbita pepo var. giromontiina*	2022	GP, Poland	HTS
**PV665990**	BOR22/9A	*Cucurbita pepo var. giromontiina*	2022	GP, Poland	HTS
**PV665991**	BOR22/15S	*Cucurbita pepo var. giromontiina*	2022	GP, Poland	HTS
**PV665992**	BOR22/18S	*Cucurbita pepo var. giromontiina*	2022	GP, Poland	HTS
**PV665993**	BOR22/19S	*Cucurbita pepo var. giromontiina*	2022	GP, Poland	HTS
**PV665994**	BOR22/20A	*Cucurbita pepo var. giromontiina*	2022	GP, Poland	HTS
**PV665995**	GN22/G12	*Cucurbita pepo var. giromontiina*	2022	KP, Poland	HTS
**PV665996**	GN22/G19	*Cucurbita pepo var. giromontiina*	2022	KP, Poland	HTS
**PV665997**	GN22/G21	*Cucurbita pepo var. giromontiina*	2022	KP, Poland	HTS
**PV665998**	GN22/G29	*Cucurbita pepo var. giromontiina*	2022	KP, Poland	HTS
**PV665999**	GN22/G30	*Cucurbita pepo var. giromontiina*	2022	KP, Poland	HTS
**PV666000**	GN22/G31	*Cucurbita pepo var. giromontiina*	2022	KP, Poland	HTS
**PV666001**	GN22/G35	*Cucurbita pepo var. giromontiina*	2022	KP, Poland	HTS
**PV666002**	GN22/G36	*Cucurbita pepo var. giromontiina*	2022	KP, Poland	HTS
**PV666003**	GN23/G2/4	*Cucurbita pepo var. giromontiina*	2023	KP, Poland	HTS
**PV666004**	GN23/G2/5	*Cucurbita pepo var. giromontiina*	2023	KP, Poland	HTS
**PV666005**	GN23/G2/8	*Cucurbita pepo var. giromontiina*	2023	KP, Poland	HTS

Accession no.	Isolate	Host	Collection date	Region	Sequencing method
**PV666006**	GN23/G2/13	*Cucurbita pepo var. giromontiina*	2023	KP, Poland	HTS
**PV666007**	BOR23/B3/17	*Cucurbita pepo var. giromontiina*	2023	GP, Poland	HTS
**PV666008**	BOR23/B3/18	*Cucurbita pepo var. giromontiina*	2023	GP, Poland	HTS
**PV666009**	BOR23/B3/19	*Cucurbita pepo var. giromontiina*	2023	GP, Poland	HTS
**PV666010**	BOR23/B3/20	*Cucurbita pepo var. giromontiina*	2023	GP, Poland	HTS
**PV666011**	BOR23/B3/29	*Cucurbita pepo var. giromontiina*	2023	GP, Poland	HTS
**PV666012**	BOR23/B3/1	*Cucurbita pepo var. giromontiina*	2023	GP, Poland	HTS
**PV666013**	BOR23/B3/3	*Cucurbita pepo var. giromontiina*	2023	GP, Poland	HTS
**PV666014**	BOR23/B3/8	*Cucurbita pepo var. giromontiina*	2023	GP, Poland	HTS
**PV666015**	GN23/G2/1	*Cucurbita pepo var. giromontiina*	2023	KP, Poland	HTS
**PV666016**	GN23/G2/2	*Cucurbita pepo var. giromontiina*	2023	KP, Poland	HTS
**PV666017**	GN23/G2/3	*Cucurbita pepo var. giromontiina*	2023	KP, Poland	HTS
**PV666018**	BOR23/B3/26	*Cucurbita pepo var. giromontiina*	2023	GP, Poland	HTS
**PV666019**	GN23/G2/9	*Cucurbita pepo var. giromontiina*	2023	KP, Poland	HTS
**PV666020**	BOR24/B3/20	*Cucurbita pepo var. giromontiina*	2024	GP, Poland	HTS
**PV666021**	BOR24/B3/21	*Cucurbita pepo var. giromontiina*	2024	GP, Poland	HTS
**PV666022**	BOR24/B3/12	*Cucurbita pepo var. giromontiina*	2024	GP, Poland	HTS
**PV666023**	BOR24/B3/10	*Cucurbita pepo var. giromontiina*	2024	GP, Poland	HTS
**PV666024**	BOR24/B3/23	*Cucurbita pepo var. giromontiina*	2024	GP, Poland	HTS
**PV666025**	BOR24/B3/19	*Cucurbita pepo var. giromontiina*	2024	GP, Poland	HTS
**PV666026**	BOR24/B3/16	*Cucurbita pepo var. giromontiina*	2024	GP, Poland	HTS
**PV666027**	BOR24/B3/22	*Cucurbita pepo var. giromontiina*	2024	GP, Poland	HTS
**PV666028**	BOR24/B3/13	*Cucurbita pepo var. giromontiina*	2024	GP, Poland	HTS

Accession no.	Isolate	Host	Collection date	Region	Sequencing method
**PV666029**	BOR24/B3/15	*Cucurbita pepo var. giromontiina*	2024	GP, Poland	HTS
**PV666030**	BOR24/B3/14	*Cucurbita pepo var. giromontiina*	2024	GP, Poland	HTS
**PV666031**	BOR24/B3/24	*Cucurbita pepo var. giromontiina*	2024	GP, Poland	HTS
**PV666032**	GN24/G1/2	*Cucurbita pepo var. giromontiina*	2024	KP, Poland	HTS
**PV666033**	GN24/G2/27	*Cucurbita pepo var. giromontiina*	2024	KP, Poland	HTS
**PV666034**	GN24/G2/28	*Cucurbita pepo var. giromontiina*	2024	KP, Poland	HTS
**PV666035**	GN24/G2/31	*Cucurbita pepo var. giromontiina*	2024	KP, Poland	HTS
**PV666036**	GN24/G2/20	*Cucurbita pepo var. giromontiina*	2024	KP, Poland	HTS
**PV666037**	GN24/G2/21	*Cucurbita pepo var. giromontiina*	2024	KP, Poland	HTS
**PV666038**	GN24/G2/22	*Cucurbita pepo var. giromontiina*	2024	KP, Poland	HTS
**PV666039**	GN24/G2/26	*Cucurbita pepo var. giromontiina*	2024	KP, Poland	HTS
**PV666040**	GN24/G2/33	*Cucurbita pepo var. giromontiina*	2024	KP, Poland	HTS
**PV666041**	BOR24/B3/11	*Cucurbita pepo var. giromontiina*	2024	KP, Poland	HTS
**PV666042**	BOR24/B3/18	*Cucurbita pepo var. giromontiina*	2024	KP, Poland	HTS
**PV666043**	BOR24/B3/25	*Cucurbita pepo var. giromontiina*	2024	GP, Poland	HTS
**PV666044**	GN24/G2/16	*Geranium pusillum*	2024	KP, Poland	HTS
**PV666045**	BOR24/B4/15	*Geranium pusillum*	2024	GP, Poland	HTS
**PV666046**	GN24/G2/15	*Geranium pusillum*	2024	KP, Poland	HTS

Table S2. Polish isolates of WMV, with the same host, origin and collection data, which CP sequences shows 100 % nucleotide identity.

Name of isolate used in analyses	Name of isolates excluded from analyses
GN22/G10	GN22/G17, GN22/G24, GN22/G5, GN22/G6, GN22/G7, GN22/G20, GN22/G22, GN22/G23, GN22/G1, GN22/G2, GN22/G3
GN22/G32	GN22/G25, GN22/G26, GN22/G27
GN23/G2/2	GN23/G2/3, GN23/G2/4
B1	B134
Sk4	Sk1
GN22/G33	GN22/G34, GN22/G35, GN22/G36
BOR22/B11A	BOR22/B17A, BOR22/9A
BOR22/B12S	BOR22/B14A, BOR22/B16A, BOR22/2S
BOR22/B13S	BOR22/B10A, BOR22/B36, BOR22/8S
BOR23/B3/8	BOR23/B3/19, BOR23/B3/20, BOR23/B3/29, BOR23/B3/1, BOR23/B3/3, BOR23/B3/17 , BOR23/B3/26
BOR24/B3/12	BOR24/B3/23, BOR24/B3/19, BOR24/B3/16, BOR24/B3/22, BOR24/B3/15, BOR24/B3/24, BOR24/B3/11, BOR24/B3/18, BOR24/B3/25
BOR22/6A	BOR22/7A, BOR22/15S, BOR22/18S, BOR22/19S, BOR22/20A
GN22/G9	GN22/G21, GN22/G19
GN22/G29	GN22/G30, GN22/G31
GN24/G2/28	GN24/G2/26, GN24/G2/33
GN24/G2/22	GN24/G2/20, GN24/G2/21, GN24/G2/16
BOR24/B3/10	BOR24/B3/21, BOR24/B3/20 , BOR24/B3/13, BOR24/B3/14
131	132, 134, 134z, 135, 137z, 138, 139, 1310
Szcz10	Szcz7
136	133
P1	P5, P6
Miel2	Miel3, Miel5
BOR9	BOR45

Table S3. Total number of reads, reads mapped to the reference WMV genome expressed as RPHT (reads per hundred thousand of total reads in the sample), and average coverage of WMV genome presented for each HTS sample.

Sample	Total number of reads	RPHT	Average coverage
GN22/G10	66656714	3096.65	30445.09
GN22/G11	60997768	3245.57	29215.3
GN22/G17	63995508	15148.42	142906.21
GN22/G24	88970658	21775.64	285695.13
GN22/G32	65975326	9280.90	90435.57
GN22/G33	67327728	5377.50	53807.03
GN22/G34	65316094	3326.77	32289.01
GN22/G41	76673280	4.03	45.72
GN22/G49	73110462	16.67	180.41
GN22/G45	74568550	486.50	5348.57
GN22/G51	82483030	26.86	326.91
GN22/G4	65897464	1627.60	15521.06
GN22/G5	46793364	2484.19	16890.18
GN22/G6	47779538	4730.99	33125.09
GN22/G7	48596258	2185.72	15527.01
GN22/G9	49235174	1493.42	10720.05
GN22/G14	47104416	679.62	4710.88
GN22/G15	48478250	268.78	1916.03
GN22/G16	57587006	1392.89	11805.1
GN22/G20	46960590	468.89	3216.71

Sample	Total number of reads	RPHT	Average coverage
GN22/G22	54422398	793.12	6288.11
GN22/G23	48143508	469.60	3291.34
GN22/G25	52134874	3207.03	24551.49
GN22/G26	51117604	3681.81	27631.79
GN22/G27	53296050	2288.29	17880.85
BOR22/B26	48890806	8.16	58.24
GN22/G42	53281394	1612.54	12650.76
GN22/G48	35032750	768.85	3976.61
M11	59581244	7.64	60.35
GN22/G43	48137712	122.65	869.44
GN22/G47	49567906	17.79	128.61
M4	61656618	3.49	28.44
BOR22/B11A	42295182	130.21	813.35
BOR22/B12S	53810468	236.90	1878.61
BOR22/B13S	43616852	2578.49	16635.37
BOR22/B14A	40885888	681.98	4111.17
BOR22/B16A	40313752	3945.95	23495.04
BOR22/B17A	41069984	24.79	150.34
GN22/G1	12434026	33284.21	61163.57
GN22/G2	40528646	11910.00	71546.79
GN22/G3	21282358	23176.09	72908.58
BOR22/B10A	23562428	3228.02	11261.28
BOR22/B36	17152302	24291.36	61396.47

Sample	Total number of reads	RPHT	Average coverage
BOR22/2S	98063218	208.12	2021.09
BOR22/4A	92092226	13.72	124.77
BOR22/6A	86741530	788.25	6758.65
BOR22/7A	100804374	2677.91	26714.97
BOR22/8S	97469826	377.55	3636.2
BOR22/9A	85457770	679.04	5737.81
BOR22/15S	97063134	3590.63	34478.83
BOR22/18S	76747752	1948.73	14829.53
BOR22/19S	83966378	231.79	1927.14
BOR22/20A	75381668	1515.66	11314.14
GN22/G12	76322918	8343.41	63156.8
GN22/G19	74103474	4308.42	31496.74
GN22/G21	70394870	4185.06	29199.37
GN22/G29	55198308	5749.67	31444.25
GN22/G30	91458768	9091.81	82342.86
GN22/G31	85257894	3361.64	28394.47
GN22/G35	88270898	1677.85	14765.74
GN22/G36	78541926	3744.14	29350.89
GN23/G2/4	20705402	9536.32	29032.99
GN23/G2/5	42285726	6319.80	39460.25
GN23/G2/8	48976926	6981.94	50498.17
GN23/G2/13	22007984	2103.53	6801.26
BOR23/B3/17	12665482	192.71	359.33

Sample	Total number of reads	RPHT	Average coverage
BOR23/B3/18	21459472	351.77	1112.19
BOR23/B3/19	25229960	330.19	1126.41
BOR23/B3/20	12453754	206.28	375.54
BOR23/B3/29	4385770	825.99	534.09
BOR23/B3/1	4999410	209.92	154.57
BOR23/B3/3	10769998	726.37	1144.95
BOR23/B3/8	8134214	605.93	722.15
GN23/G2/1	11401284	9318.96	15579.98
GN23/G2/2	7737918	9629.13	10896.33
GN23/G2/3	25141226	5548.89	20434.69
BOR23/B3/26	3689276	757.93	413.27
GN23/G2/9	30041566	3166.52	14001.85
BOR24/B3/20	50216350	1302.00	9619.33
BOR24/B3/21	49727064	546.60	4002.02
BOR24/B3/12	49318116	1343.94	9762.96
BOR24/B3/10	52097994	2908.72	22298.92
BOR24/B3/23	49789480	1612.62	11812.11
BOR24/B3/19	48460866	756.43	5398.89
BOR24/B3/16	54167412	365.49	2906.42
BOR24/B3/22	47549658	626.91	4391.14
BOR24/B3/13	50065488	433.81	3191.1
BOR24/B3/15	50752534	1227.29	9169.69
BOR24/B3/14	50894374	627.14	4691.36

Sample	Total number of reads	RPHT	Average coverage
BOR24/B3/24	50055154	137.39	1010.86
GN24/G1/2	48441188	214.97	1531.05
GN24/G2/27	55971678	1402.98	11571.35
GN24/G2/28	48111918	2315.46	16394.57
GN24/G2/31	50827916	636.42	4727.2
GN24/G2/20	50588650	3516.75	25962.89
GN24/G2/21	49566184	9595.99	69542.13
GN24/G2/22	49871160	6750.50	49257.68
GN24/G2/26	48574578	5577.78	39756.03
GN24/G2/33	48750814	2671.39	19070.75
BOR24/B3/11	50288242	3920.87	29097.09
BOR24/B3/18	47552518	1813.25	12731.54
BOR24/B3/25	47473204	453.28	3177.66
GN24/G2/16	47648992	89.00	626.13
BOR24/B4/15	47184808	1967.93	13712.63
GN24/G2/15	49218210	66.85	485.22
